# Impacts of Local Effects and Surface Loads on the Common Mode Error Filtering in Continuous GPS Measurements in the Northwest of Yunnan Province, China

**DOI:** 10.3390/s20185408

**Published:** 2020-09-21

**Authors:** Keliang Zhang, Yuebing Wang, Weijun Gan, Shiming Liang

**Affiliations:** 1 State Key Laboratory of Earthquake Dynamics, Institute of Geology, China Earthquake Administration, Beijing 100029, China; ybwang@seis.ac.cn (Y.W.); wjgan@ies.ac.cn (W.G.); liangshiming@ies.ac.cn (S.L.); 2 Department of Geophysical Network, China Earthquake Networks Center, Beijing 100045, China

**Keywords:** continuous GPS measurements, common mode error, hydrological water storage, loading deformation, transient processes, GRACE

## Abstract

While seasonal hydrological mass loading, derived from Gravity Recovery and Climate Experiment (GRACE) measurements, shows coherent spatial patterns and is an important source for the common mode error (CME) in continuous global positioning system (cGPS) measurements in Yunnan, it is a challenge to quantify local effects and detailed changes in daily GPS measurements by using GRACE data due to its low time and spatial resolutions. In this study, we computed and compared two groups of CMEs for nine cGPS sites in the northwest Yunnan province; rCMEs were computed with the residual cGPS time series having high inter-station correlations, while oCMEs were computed with all the GPS time series. The rCMEs-filtered time series had smaller variances and larger root mean square (RMS) reductions than those that were oCMEs-filtered, and when the stations local effects were not removed, spurious transient-like signals occurred. Compared with hydrological mass loading (HYDL), its combination with non-tidal atmosphere pressure and ocean mass reached a better agreement with the CME in the vertical component, with the Nash–Sutcliffe efficiency (NSE) increasing from 0.28 to 0.55 and the RMS reduction increasing from 15.19% to 33.4%, respectively. Our results suggest that it is necessary to evaluate the inter-station correlation and remove the possible noisy stations before conducting CME filtering, and that one should carefully choose surface loading models to correct the raw cGPS time series if CME filtering is not conducted.

## 1. Introduction

Continuous GPS (cGPS) measurements not only provide precision position time series of tectonic signals (e.g., plate motion, crustal strain accumulation) [1,2,3,4] but record temporal responses to the variations of surface loading from various sources such as tidal loading, atmospheric pressure, continental water storage [5,6,7,8,9,10,11,12], and unmodeled systematic errors including the mismodelling of satellite orbits, receiver antenna phase center corrections, and multipath effects [6,13]. Both environmental responses and systematic errors will result in spatiotemporal coherent features of cGPS measurements in a rather large area that may extend to more than 2000 km [14]. If the magnitudes of such coherent features are large enough or larger than the tectonic responses, it will be a challenge to detect useful information due to the high uncertainties. As a result, such coherent features are usually called common mode errors (CMEs), which are the main spatially-correlated signals in the cGPS measurements. When CME is extracted and removed from the measurements, the remaining residuals will usually have a much better resolution and thus provide a chance to detect the transient deformation underlying the raw data [15,16,17].

In order to improve the signal-to-noise ratio of cGPS position time series, several spatial filtering methods, such as stacking [14,15,18], principal component analysis (PCA) [13], the modified version of PCA [19,20], and independent component analysis [21], have been proposed to extract the CME. For regional networks, the stacking approach works well due to the highly spatial coherence between any two stations. As an unsupervised learning method, PCA converts the original datasets into a set of linearly independent features based on the eigenvalue decomposition of the variance–covariance matrix of the data. Both stacking and PCA are suitable under the assumption that the measurements are highly correlated with each other. In practice, when the inter-station distances become larger, the spatial coherence breaks down and the common mode bias becomes progressively smaller [18]. In this case, if the CME filtering is forced to be applied to a large region, instead of filtering any actual common mode errors, it may actually lead to spurious signals to the remaining stations. Particularly, if these measurements are independent, each of them can be regarded as an independent component, so that there is no longer a need for PCA decomposition, or new measurements are required to eliminate the noises that are resulting from the common sources.

However, since the CMEs in GPS measurements differ from one region to another, there is no general criterion on how to construct CME filtering [4,6], particularly on how to set weighting factors among all the involved measurements. In practice, in order to provide a feasible and robust method for estimating CMEs on regional or global scale GPS networks, several factors have been taken into account or discarded. For example, for the weighted stacking method, Márquez-Azúa and DeMets [14] took the length of measurements and site distance as weights to estimate the daily CME. By using this algorithm, Wang et al. [22] scaled the distance-dependent weighting factor linearly from 1.0 for sites within 500 km to zero for sites farther than 2000 km. More recently, Tian and Shen [23] weighed the measurements with the interstation correlations and distances between neighboring sites in the form of the product of correlation coefficient and an inverse exponential function of squared distance. Although all these algorithms are based on the assumption that correlations are smaller for sites over large distances, or that they are affected by local-scale or site-specific variations [23], there are no detailed quantitative descriptions on how much the process of weighting factors can suppress such local effects. In comparison, in a pioneer work, Dong et al. [13] checked the spatial coherence of stations by visual inspection of the decomposed principle components, and found that the CME was strongly affected by local deformation of three stations located in the rim of the Santa Ana basin due to aquifer activities; in that case, they discarded all those sites with local effects.

Yunnan province is located in the path of monsoons moving north- and northeastward from southeast Asia, and as such it is significantly influenced by large-scale hydrological water storage. Its climate is characterized with clear wet and dry seasons. In the wet season from June to August, the monsoons bring Yunnan abundant precipitation; while in the dry season, inadequate precipitation may lead to severe drought due to the reduction of the replenishment of water storage [24]. Such seasonal fluctuations in water storage have caused large seasonal deformation of the Earth’s crust that has been recorded in cGPS measurements [25,26].

**Figure 1:**
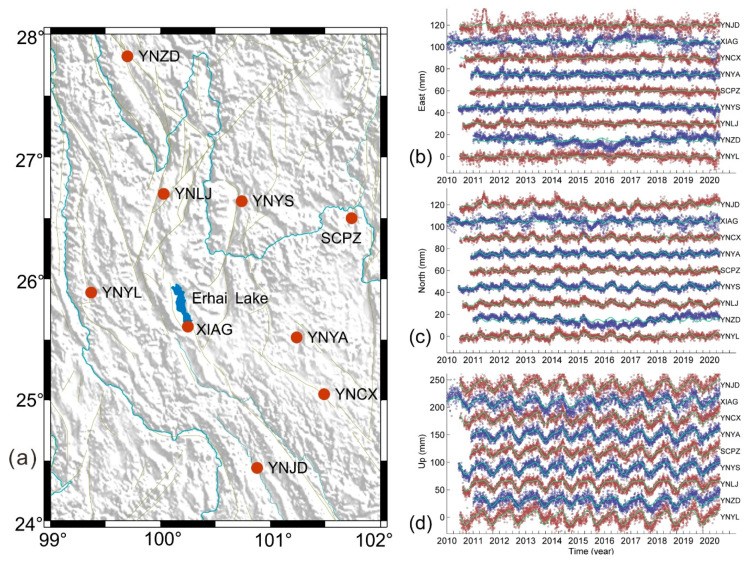
(**a**) Locations of cGPS stations (solid red circles) in the northwest Yunnan, and detrended GPS position time series for (**b**) east, (**c**) north, and (**d**) vertical components, respectively. The green curves show the seasonal variations modeled with annual and semi-annual harmonics.

Recent studies have shown good consistency between seasonal variations in cGPS vertical time series and those modeled with water storage variations derived from the Gravity Recovery and Climate Experiment (GRACE) datasets [24,27,28,29]. Although these studies have provided a better understanding of these highly temporal cGPS time series correlated with CME filtering [19,27,28,29], their inferences on the consistency between cGPS time series and hydrological mass loading are based on monthly GRACE measurements. For example, Sheng et al. [27] found that, at a monthly time scale, the hydrological loading deformation accounts for a major percentage for the CME of cGPS vertical time series, nevertheless, the time span of measurements for 12 stations was no more than 2 years. Hao et al. [28] used the annual and semiannual sine function modeled from monthly GRACE data to remove the atmospheric and hydrological seasonal loading in daily cGPS measurements. In fact, more evidence has suggested that the annual signals in cGPS time series are a response to variations in the Earth’s surface mass loading [6,24,29], including changes of non-tidal atmospheric pressure, ocean loading, and hydrological water storage. Furthermore, in these previous studies, the time series of the vertical component were occupied to obtain the CME without ignoring any weakly correlated time series. Taking XIAG (Figure 1a) as an example, XIAG station is located on the south bank of Erhai Lake, which is the second largest reservoir in Yunnan province; the water level fluctuates annually with an amplitude about 3 m and has resulted in significant anomalies in gravity measurements [30]. Such loading effects associated with large local hydrological water storage may further result in significant responses in cGPS measurements [27]. Nevertheless, both the monthly time interval and ~400 km spatial resolution of GRACE data are too low to discern these local effects or the detailed changes in daily GPS measurements [24,27,28]. Therefore, it is necessary to quantitatively evaluate the impacts of local effects and loading deformation on the CME filtering for daily cGPS measurements.

For this purpose, as a case study, we took nine cGPS stations in the northwest of Yunnan Province into account to stress the importance of considering the local effects before CME. We first evaluated the correlations among the position time series of these cGPS measurements. With the highly correlated seven stations as a supervised group, we obtained the CMEs time series by weighted stacking for three components of these stations, and then the supervised results were compared with the unsupervised one. Finally, we compared the CMEs with the modeled daily loading displacements, induced by non-tidal atmospheric and oceanic pressures, and surface hydrological mass variations. Our results suggest that it is necessary to evaluate the inter-station correlation and remove the possible noisy stations before conducting CME filtering, and that one should carefully choose surface loading models to correct the raw cGPS time series instead of using CME filtering.

## 2. Materials and Methods

### 2.1. Processing of GPS Datasets and Post-Processing of the GPS Residual Daily Time Series

Daily vertical displacements of nine cGPS stations (Figure 1) from the Crustal Movement Observation Network of China (CMONOC) were processed with GAMIT/GLOBK software [31] in an ITRF2008 reference frame [2]. In the processing, we used the VMF1 tropospheric mapping function to estimate the hydrostatic and wet zenith delays. The effects from polar motion, solid Earth tides, and ocean tides were removed in the data processing. The XIAG site has been continuously observed since the late 1990s, while the other stations have been continuously measured since mid-2010 and/or mid-2011. The locations of all the stations are shown in Figure 1 and Table 1.

In the tectonic background, the long-term rates of three components of a continuous GPS station remain constant. For simplification, a linear rate $v_{0}$ and initial position $x_{0}$ are estimated with least squares regression, while the seasonal terms are retained as we regard the common periodicity as a CME. The residuals’ time series of each component r(t) can be obtained by removing the linear trend from the measurements.

(1) $$r\left(t\right)\;=\;x\left(t\right){\;-\;x}_{0}{\;-\;v}_{0}\left({t\;-\;t}_{0}\right)\;\varepsilon \;$$

where t is the time and $t_{0}$ the origin of time, $x_{0}$ is the initial coordinate at time $t\;=\;t_{0}$, $v_{0}$ is a constant, the linear velocity of the point, and ε is the noise term. The noise term is observational white noise$\;\varepsilon \sim N\left(0,\sigma^{2}\right)$, where$\;\sigma^{2}$ is the error variance of daily solution.

We applied the median-interquartile range (IQR) algorithm [16] to detect and remove the outliers in the postfit residuals. In this cleaning process, the IQR of a dataset is defined as the difference between its 75th and 25th percentiles, the values are outliers when the absolute values of difference between the dataset and its median are larger than three times the IQR. The postfit residual position time series are cleaned separately in the east, north, and vertical (up) directions.

### 2.2. Loading Deformation Due to Atmosphere, Non-Tidal Ocean, and Hydrological Water Mass

For each GPS station, we obtained the daily elastic displacement time series resulting from surface loads of non-tidal atmospheric and oceanic pressure and hydrological mass variations with gridded loading displacements stored on a regular 0.5° × 0.5° global grid with 24 h hydrological water storage (HYDL) and 3 h non-tidal atmospheric pressure (NTAL) and ocean loading (NTOL) provided by the GeoForschungsZentrum (GFZ) loading service (https://isdc.gfz-potsdam.de/esmdata/). The elastic surface deformations are calculated in the spatial domain by convolving the loading of Green’s function in the center of the Earth’s figure frame (CF) with the loaded Love numbers using modelled mass distributions from the models ECMWF (European Center for Medium-Range Weather Forecasts), MPIOM (Max-Planck-Institute for Meteorology Ocean Model), and Land Surface Discharge Model [32]. Please refer to Dill and Dobslaw [32] and the Geo Forschungs Zentrum (GFZ) loading service (https://isdc.gfz-potsdam.de/esmdata/) for more detailed information about the loading deformation datasets and mass distribution models.

### 2.3. Evaluation of Similarity among GPS Time Series

The Pearson’s correlation coefficients (*R*) is used to measure the statistical relationship between two measurements.

(2) $$R\;=\;\frac{\sum_{i=1}^{N}\left(y_{p}{(i)\;-\bar{y}}_{p}{)(y}_{q}{(i)\;-\bar{y}}_{q}\right)}{\sqrt{\sum_{i=1}^{N}{\left(y_{p}(i)\;-{\bar{y}}_{p}\right)}^{2}}\sqrt{\sum_{i=1}^{N}{\left(y_{q}{(i)\;-\bar{y}}_{q}\right)}^{2}}}\;$$

where $y_{p}\left(i\right)$ and $y_{q}\left(i\right)$ are the values of *p*-th and *q*-th measurements at the *i*-th epoch, with mean values of ${\bar{y}}_{p}$ and ${\bar{y}}_{q}$, respectively. *N* is the time length of observations. The *R* values of 1 and −1 denote a perfect positive and negative correlation between two measurements, respectively.

Because the correlation coefficient value provides no information about the magnitude discrepancy between the two datasets, we further computed the Nash–Sutcliffe Efficiency (NSE) [33] to evaluate the consistency between CMEs and loading deformation. The NSE is a function to quantitatively evaluate the amplitude discrepancy, which indicates how well the observed data fits the simulated data. NSE ranges from infinity to 1. When NSE = 1, it means that these two variables have perfect consistency; when NSE = 0, it indicates that simulated data are equivalent with the mean of the observation data. Whereas if the NSE value is less than 0, the discrepancy of amplitudes between two variables becomes unacceptable.

(3) $$\mathrm{NSE}\;=\;1\;-\;\frac{\sum_{i=1}^{N}{\left(y_{\mathrm{pred}}{(i)\;-\;y}_{\mathrm{obs}}\left(i\right)\right)}^{2}}{\sum_{i=1}^{N}{\left(y_{\mathrm{obs}}{(i)\;-\bar{y}}_{\mathrm{obs}}\right)}^{2}}\;$$

where $y_{obs}\left(i\right)$ and $y_{pred}\left(i\right)$ are the values of the *i*-th observation and prediction, respectively; ${\bar{y}}_{obs}$ and ${\bar{y}}_{pred}$ are the mean values of observation and prediction, respectively, and *N* is the number of stations.

### 2.4. Estimate of the CMEs in cGPS Position Time Series

When the correlation coefficients are larger than 0.5, the postfit residuals are then stacked to compute the CME of each component by weighting with the inverse of the error variance of the daily solutions.

(4) $$\mathrm{CME}\;=\;\frac{\sum_{i=1}^{N}\sum_{j=1}^{Nt}\frac{r_{i,j}}{\sigma_{i,j}^{2}}}{\sum_{i=1}^{N}\sum_{j=1}^{Nt}\frac{1}{\sigma_{i,j}^{2}}}\;$$

where$\;r_{i,j}$ and $\sigma_{i,j}^{2}$ are the values of residuals and error variance of daily solutions of *i*-th station at *j*-th epoch, respectively.

In order to test whether the low correlated station will affect the CMEs, we set two groups of stations to compute the CMEs for all three components. In the first group, all nine stations were involved in computation of CMEs. While in the second group, only the seven stations that showed high inter-correlation were selected. Hereinafter, the CMEs derived from the first and the second groups are referred to as oCMEs and rCMEs, respectively.

### 2.5. Evaluation of CMEs

For the purpose of evaluating the reliability of these two CME corrections, we calculated the variance of the corrected time series for the east, north, and up components. The lower value of variance of the corrected time series indicates a better correction.

The F-test was applied to evaluate which variance was smaller for the postfit residuals after correction of oCMEs and rCMEs. First, we set the null hypothesis H0 = 0 by assuming these two variances are statistically equal to each other. The F-statistic is the ratio of two variances, i.e., $F\;={\;\sigma }_{\mathrm{rCME}}^{2}/\sigma_{\mathrm{oCME}}^{2}$, where $\sigma_{\mathrm{rCME}}^{2}$ and $\sigma_{\mathrm{oCME}}^{2}$ are variances for the residual time series after rCME and oCME corrections. If the F-statistic is larger than the upper critical value or less than the lower critical value, the null hypothesis is false; and meanwhile if the *p*-value is also less than the significant level, e.g., 5%, then we will reject the null hypothesis. In this case, if the F-statistic is less than the upper critical value, the variance of the numerator is less than that of the denominator; whereas if the F-statistic is larger than the lower critical value, the variance of the numerator is larger than that of denominator. Accordingly, we can make a decision to select the better result to correct the original GPS time series.

Furthermore, the RMS reduction is also used to evaluate how well the CME fits the residual position time series.

(5) $$\mathrm{RMS}\%\;=\;\frac{{\mathrm{RMS}}_{\mathrm{GPS}}{\;-\;\mathrm{RMS}}_{\mathrm{GPS}-\mathrm{CME}}}{{\mathrm{RMS}}_{\mathrm{GPS}}}\;$$

where $RMS_{\mathrm{GPS}}$ and $RMS_{\mathrm{GPS}-\mathrm{CME}}$ are the RMSs of the residual position time series and the CMEs for three components, respectively. RMS reduction can also be used to test what percentage the loading deformation contributes to the CME, in which case the subscript GPS and CME can be replaced with CME and Loading, respectively.

## 3. Results

### 3.1. Residual cGPS Time Series

The residual time series of three components, after removal of a best fitting linear trend, are shown in Figure 1 for all nine cGPS stations. For the up component, the residual time series show strong annual and semi-annual variations at all stations. Table 1 shows the amplitudes of annual and semi-annual variations of the three components. It is notable that, for XIAG and YNJD, the residual time series of the east and north components are much more scattered than those of all the other stations. Furthermore, there are abnormal transient-like signals in both the east and north components of XIAG during the period from 2015 to 2016. As such, these two stations were suspected to be significantly affected by local effects, and the reliability in the CME corrections may be also vulnerable. We further computed two types of CMEs by evaluating the inter-station correlation and show the difference between these two CME corrections.

### 3.2. Inter-Station Correlation

**Figure 2:**
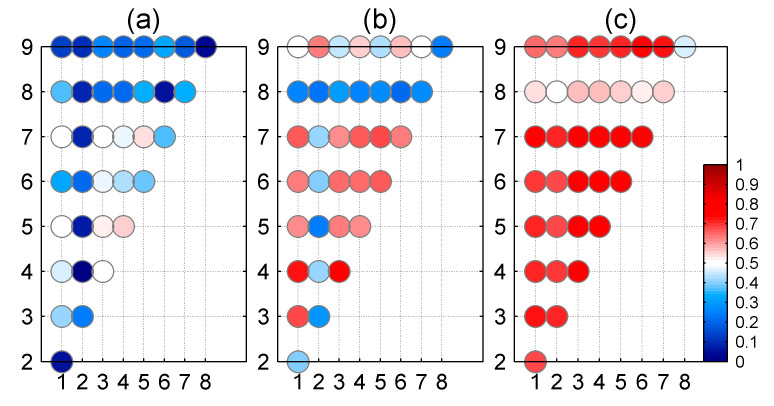
Correlation coefficients of (**a**) east, (**b**) north, and (**c**) up components for any two GPS stations. The numbers labeling the axis denote the stations listed in Table 1.

Figure 2 and Table 1 show the inter-station correlation coefficients of three (east, north, and up) components for 36 GPS station pairs. The correlation coefficients are relatively low for the north and east components. For the east component, only five station pairs show correlation coefficients larger than 0.5. For the north component, the correlation coefficients are greater than 0.5 for 19 station pairs. In comparison, the up component shows larger correlation coefficients, with 34 station pairs showing correlation coefficients larger than 0.5, and there are more than 26 station pairs with correlation coefficients larger than 0.65.

**Figure 3:**
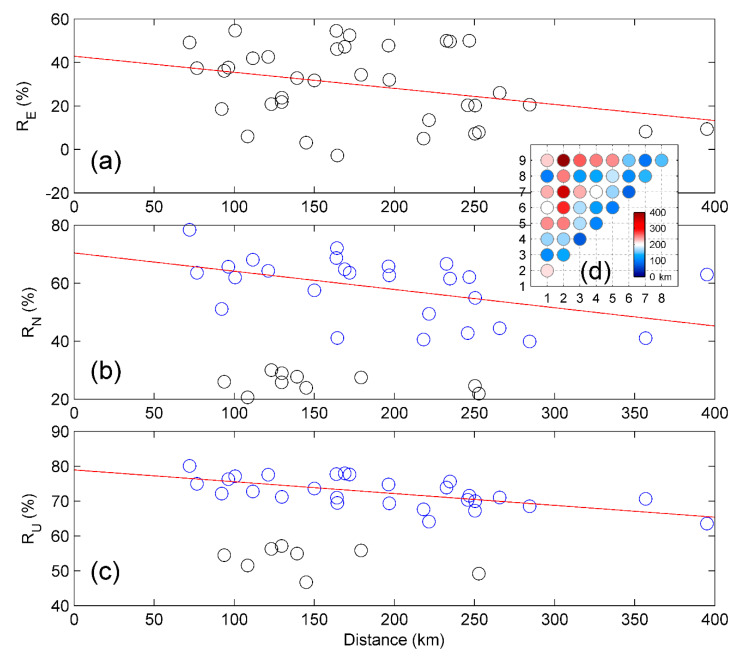
Inter-station distance vs. correlation coefficients for (**a**) east, (**b**) north, (**c**) up components, and (**d**) the inter-station distances. The circles in blue show that the correlation coefficients decrease as the distances increase; while the circles in gray denote there is no apparent linear correlation.

Figure 3 shows that the correlation coefficients decrease with the increasing inter-station distances. Although Figure 3a shows an overall negative linear trend, the variance is too large to be expressed as a linear trend. While after ignoring the low coefficients, both north and up components show linear trends with correlation coefficients decreasing with increasing distances. This confirms that the correlation coefficient becomes smaller when the sub-network is extended over larger areas.

### 3.3. Comparison between the oCMEs and rCMEs

**Figure 4:**
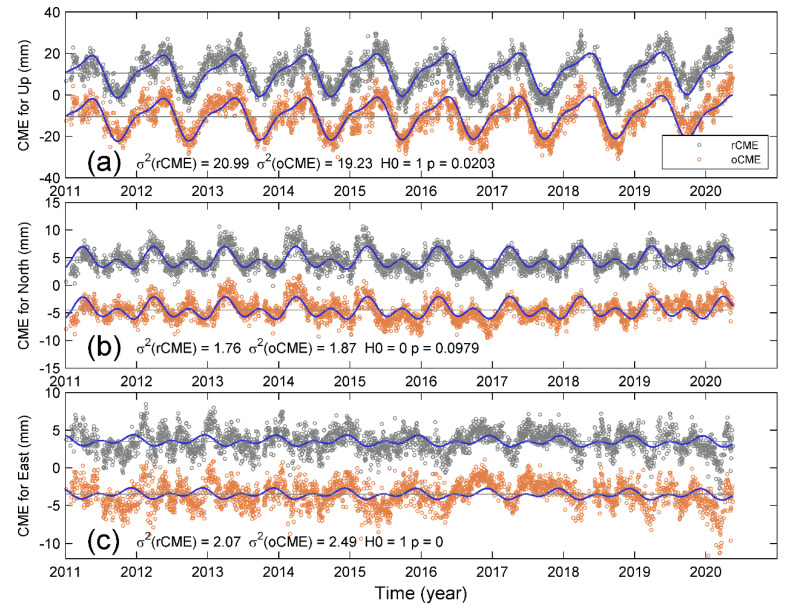
Comparison between three components of oCMEs and rCMEs. Note that offsets of ±3.5, ±4.5, and ±10.5 were added to the oCMEs and rCMEs for (**a**) up, (**b**) north, and (**c**) east components, respectively. The open circles in gray and yellow denote rCME and oCME, respectively. The curves in blue show the modeled annual and semi-annual variations.

Figure 4a–c shows the oCMEs and rCMEs for three components, respectively. In order to display and distinguish between oCMEs and rCMEs more easily, we add an offset to each component; the offsets were ±3.5, ±4.5, and ±10.5 for east, north, and up components, respectively; also shown are the modeled annual and semi-annual variations in blue curves, which show that the vertical CMEs have the most remarkable seasonal variations, the north CMEs the second highest variation, while the east CMEs have minor seasonality. Such discrepancies further reflect the different inter-station correlations for the three-component displacement time series. Because of the similarities in both amplitude and phase for each component of oCMEs and rCMEs, it is not easy to distinguish between them by visual inspection. We computed the variances of postfit residuals after rCMEs and oCMEs filtering, i.e., $\sigma^{2}\left(\mathrm{rCME}\right)$ and $\sigma^{2}\left(\mathrm{oCME}\right)$ after removing a combination of linear trend and annual and semi-annual variations. Our calculations show that the east and up components are significantly different based on the F-test and *p*-values at the significant level of 5% (Figure 4a,c). Nevertheless, the postfit variances of rCME and oCME for the north component are statistically the same based on both F-test and *p*-value, with a value larger than 0.05 (Figure 4b).

### 3.4. CME Corrections of GPS Position Time Series

**Figure 5:**
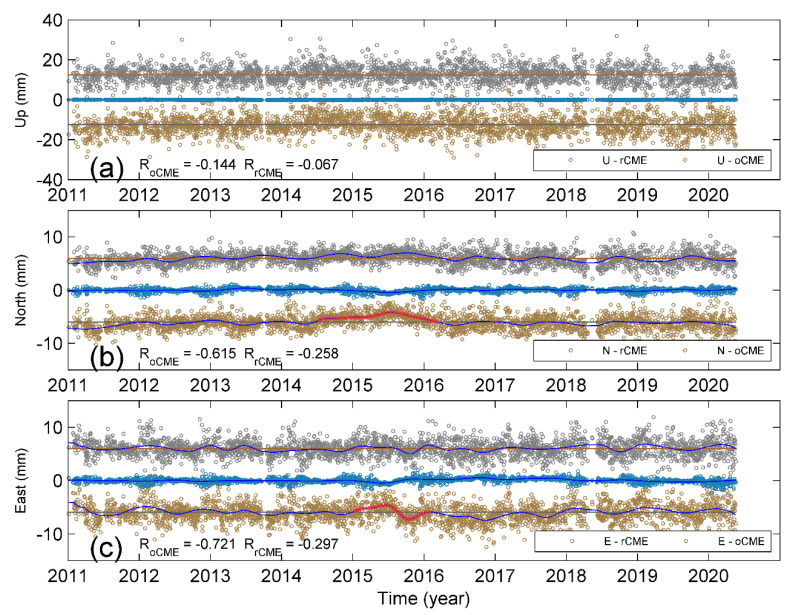
Comparison between rCME correction (gray circles) and oCME correction (yellow circles) for (**a**) up, (**b**) north, and (**c**) east components of YNYA; also shown are the weighted residuals (blue circles) of three components of XIAG, which are weakly correlated with all the other stations. The curves in blue are non-linear trends. The $R_{\mathrm{oCME}}$ and $R_{\mathrm{rCME}}$ are correlation coefficients between residual time series of XIAG and that after oCME and rCME corrections of YNYA. The dashed red curves in oCME-corrected north and east components denote the spurious transient-like signals.

We corrected the GPS time series for three components with oCMEs and rCMEs. We took YNYA as an example to show the differences between oCME correction (yellow circles) and rCME correction (gray circles) for east, north, and up components (Figure 5). On the whole, the oCME-corrected time series are more scattered than those of the rCME correction. More specifically, after rCMEs correction, the variances of filtered time series of east, north, and up components decreased from 3.9 mm^2^, 2.9 mm^2^, and 36.1 mm^2^ to 2.2 mm^2^, 1.3 mm^2^, and 17.1 mm^2^, respectively, which are statistically smaller than those with oCME correction (Table 2).

**Figure 6:**
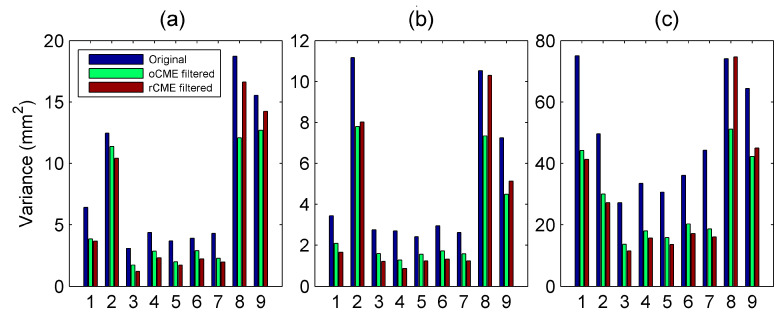
The variances of postfit residuals for (**a**) east, (**b**) north, and (**c**) up components. The bars in blue, green, and red denote variances of the postfit residuals for the original time series and the time series after oCME and rCME filtering, respectively. Except for XIAG and YNYL, most of the variances after rCME correction are less than those corrected with oCME for all three components. The numbers labeling the horizontal axis denote the stations listed in Table 1.

Table 2 and Figure 6 show the variances of postfit residuals of east, north, and up components for all nine cGPS stations. In Figure 6, the bars in blue, green, and red denote variances of the postfit residuals for the original time series and the corrected time series with oCME and rCME, respectively. The F-test was also applied to evaluate whether the variance after rCMEs correction was less than that of the oCME correction. Except for XIAG and YNYL, nearly all the other seven stations showed obvious reduction of variances of postfit residuals for all three components when rCMEs were used instead of oCMEs. Four exceptions of statistically equal variances were for the east and up components of YNYL, the north component of YNZD, and the up component of YNJD (Table 2, Figure 6).

Furthermore, the RMS reductions of oCMEs and rCMEs for all three components are shown in Table 3. It shows that when rCMEs are used instead of oCMEs, the RMS reductions are increased for the three components of almost all the highly inter-correlated seven stations except for YNZD, whose RMS reduction of oCME is slightly larger than that of rCME in the north component.

### 3.5. Comparison rCMEs with Loading Deformation

**Figure 7:**
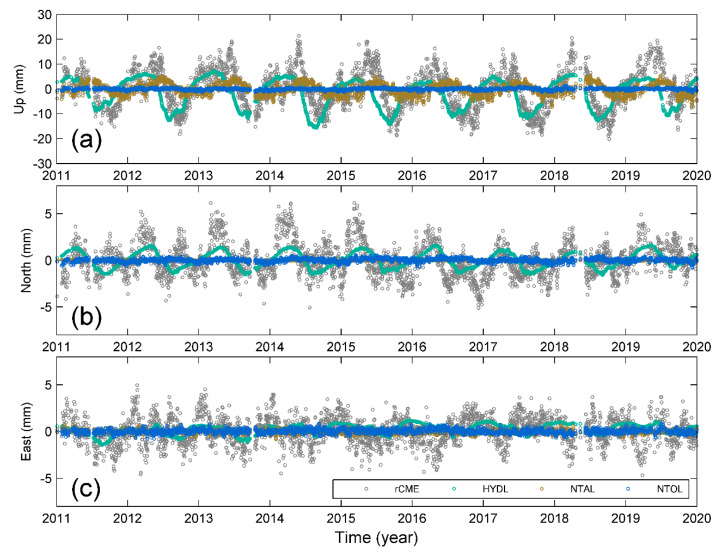
Comparison between rCMEs and loading deformations in response to HYDL, NTAL, and NTOL for (**a**) up, (**b**) north, and (**c**) east components, respectively. The open circles in gray, cyan, yellow and blue denote rCME, HYDL, NTAL and NTOL, respectively.

Figure 7 shows rCMEs (gray open circles) stacked from the highly correlated residual position time series; the annual amplitudes are 0.45 mm, 1.05 mm, and 8.70 mm for the east, north, and up components, respectively. The stacked displacements are loaded from HYDL, NTAL, and NTOL. In these three loading terms, the HYDL loadings have the largest annual amplitudes of 0.57 mm, 1.15 mm, and 7.28 mm for the east, north, and up components, respectively. In comparison, the effects due to the non-tidal atmosphere and ocean are minor, with the annual amplitudes of 2.61 mm and 0.23 mm in vertical displacements, respectively, while the annual amplitudes are less than 0.1 mm for both east and north components.

For each loading term, the NSE with the rCMEs and the corresponding RMS reductions are listed in Table 4. It is notable that there are apparent phase lags between the loading responses and the rCMEs of the three components. As a result, neither the RMS reduction nor the NSE show significant agreement between the loading effect and rCMEs. Although the HYDL loading can contribute a 15.19% RMS reduction to the CME of the up component, it is relatively low compared with the consistency of their annual amplitudes.

**Figure 8:**
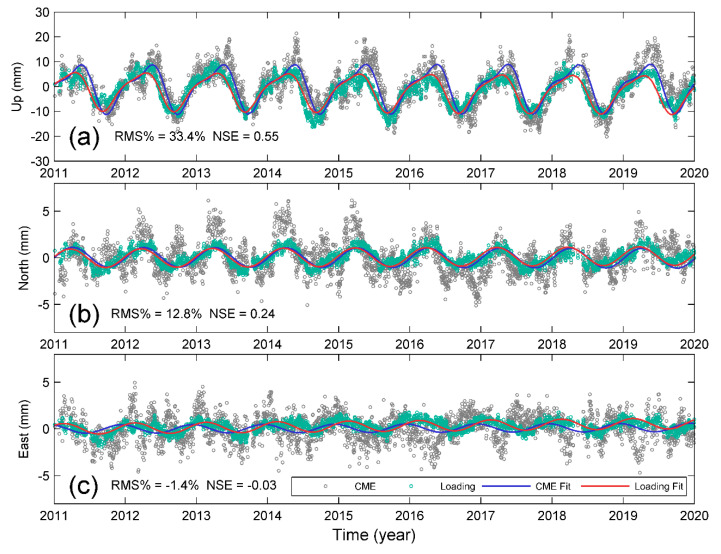
Comparison between rCMEs and combined loading deformation for (**a**) up, (**b**) north, and (**c**) east components, respectively. The loading deformation is a combination of elastic response to NTAL, NTOL, and HYDL provided by Dill and Dobslaw [32]. The RMS% denotes the RMS reduction for each component. The curves in blue and red denote the modeled annual and semi-annual variations for rCMEs and loading deformation, respectively.

We thus compared the rCMEs and combinations of these three loading terms. Figure 8 shows both rCMEs (gray open circles) and the combined loading deformation (green open circles) and the modeled annual and semi-annual variations for three components. As expected, the combined loading deformation in the up component has the most remarkable seasonal variations and agrees much better with the CME than before combination; the NSE increases from 0.28 to 0.55, and RMS reduction increases from 15.19% to 33.4% (Table 4). In contrast, the phase lag in the east component is too large to reach a good agreement between them, resulting in both a negative NSE and RMS reduction. For the north component, the increases in both NSE and RMS reduction are also minor.

## 4. Discussions

In this study, we chose seven sites that show high inter-station correlations to conduct CME filtering for a local network and discarded the other two sites to avoid any possible bias due to misleading weighting factors. Figure 6 and Table 2 show that the rCME filtering is much better than that of the oCMEs in which the low-correlated sites are included. Since XIAG is weakly correlated with all the other stations, we further examined if there are any abnormal signals when the residual time series of XIAG are involved. For each component, we estimated the non-linear trends (curves in blue) for the two corrected time series and the weighted residuals (blue circles) of XIAG, and then computed the correlation coefficients between the weighted time series of XIAG and these two corrections. Figure 6a shows a weak correlation in the up component. In comparison, Figure 6a,b show that the weighted residual time series of XIAG are negatively correlated with the oCME correction in both the east and north components with coefficients of −0.732 and −0.626, respectively, indicating a large percent of oCME correction is contributed from the weighted residual time series of these two components of XIAG. Furthermore, compared with the rCME correction, a transient-like signal is present in the oCME corrections during the period from summer 2014 to the beginning of 2016 in the north component (Figure 5b). During the time from 2015 to 2016, a similar spurious transient is present in the oCME-corrected east component (Figure 5c). Although transient deformation may be present due to the intense drought [10,24], spurious transients will lead to misunderstanding of the physical processes.

Our results suggest that if any individual station affected by local effects is involved in the CME correction, spurious transient deformation may be present in the filtered time series (Figure 5). It is notable that even for such a regional network, whose largest inter-station distance is less than 400 km, the supervised model, by simply ignoring sites with low inter-station correlation, it is possible to avoid bias from local effects and also make a significant reduction of error in the corrected residual time series.

Regardless of the bias from local effects of low-correlated sites, our results of rCMEs and oCMEs show remarkable annual and semi-annual variations (Figure 4, Figure 7 and Figure 8), particularly in the up and north components. Several previous studies have suggested that the seasonal variations in the up component of cGPS measurements in Yunnan are mainly caused by the hydrological mass redistribution, which has also been detected from GRACE gravity measurements [22,24,27,28,29]. Such inference is physically reliable because the Yunnan province is located in the margin of the South Asian monsoon; the large amount of precipitation during the summer monsoon season will cause a significant deflection of the Earth’s surface. Every year, the monsoon season occurs from June through September, nevertheless, the vertical displacements reach the lowest trough in October, forming a phase lag as shown in Figure 8. In comparison, Figure 8a shows that the atmospheric pressure applied on the surface has a phase lag later than that of the surface deformation. As a result, although the hydrological mass loading has the largest contribution to the magnitude, only when combined with NTAL and NTOL loadings does the combined loading deformation reach a good agreement with the CME (NSE = 0.55). Such agreement suggests that the seasonal variations in CME result from entire surface loading rather than the hydrological mass itself.

Such large-scale seasonal hydrological mass variations associated with the South Asian monsoon have caused seasonal deformations with larger annual amplitudes recorded in cGPS measurements in the Himalayas and Southeast Asia [8,9,11,34]. However, there are several sites showing mismatches with the loading deformation because of local effects such as groundwater irrigation [9] as well as flood [34]. These studies suggest that the cGPS measurements in horizontal directions are much more sensitive to the shorter spatial wavelengths of regional loads and local effects [27]. As such, a loading deformation model based on in situ measurements or surface mass redistribution of finer resolution is required to study any individual local effects.

## 5. Conclusions

In this study, we computed and compared two sets of CMEs for a local cGPS network in Norhtwest Yunnan province, China; the rCMEs were computed with the residual time series having high inter-station correlations, while a second CME, oCMEs, used all the residual time series. Our results show that after rCMEs filtering, the corrected residual time series have smaller variances and larger RMS reductions with respect to the original residual time series than those obtained using oCME filtering, indicating that rCMEs filtering is more efficient than oCMEs filtering. Since the local effects may be present in the low correlated stations, when the oCME filtering is applied, the filtered results show transient-like signals in both the east and north components. These results suggest that if any individual stations affected by local effects are involved in the CME correction, spurious transient deformation may be present in the filtered time series. As a result, it is necessary to evaluate the inter-station correlation and remove the possible noisy stations before conducting CME filtering.

We further compared the rCMEs with loading deformation induced by hydrological mass redistribution and non-tidal atmosphere pressure and ocean mass. The results showed that the combined loading deformation in the up component had the most remarkable seasonal variations, and only when these three loading terms were combined could the loading deformation in the up component reach a good agreement with the CME. Compared with HYDL itself, the NSE and RMS reduction with combined loading increased from 0.28 to 0.55 and from 15.19% to 33.4%, respectively, suggesting that the CME of the up component is mainly the result of the combined surface loading rather than hydrological water storage itself. Nevertheless, the loading deformation does not agree well with rCMEs of the east and north components, mostly because of the spatial resolution difference between these two types of measurements. In this case, one should carefully choose surface loading models to correct the raw cGPS time series if CME filtering is not conducted.

## Figures and Tables

**Table 1:** The locations of the continuous global positioning system (cGPS) stations and the amplitudes for modeled annual and semi-annual variations.

Station List	Station Name	Location		Annual Amplitude (mm)			Semi-Annual Amplitude (mm)		
		Long (°E)	Lat (°N)	Up	North	East	Up	North	East
1	YNYL	99.37	25.89	10.9 ± 0.4	1.5 ± 0.1	0.5 ± 0.1	4.4 ± 0.4	1.3 ± 0.1	0.7 ± 0.1
2	YNZD	99.70	27.82	7.8 ± 0.3	0.9 ± 0.2	1.4 ± 0.2	2.9 ± 0.3	1.5 ± 0.2	0.4 ± 0.2
3	YNLJ	100.03	26.7	8.8 ± 0.2	1.8 ± 0.1	0.8 ± 0.1	3.4 ± 0.2	1.0 ± 0.1	0.4 ± 0.1
4	YNYS	100.75	26.68	10.1 ± 0.3	2.1 ± 0.1	0.5 ± 0.1	2.7 ± 0.3	1.0 ± 0.1	0.5 ± 0.1
5	SCPZ	101.74	26.5	8.5 ± 0.3	0.6 ± 0.1	0.3 ± 0.1	2.8 ± 0.3	1.3 ± 0.1	0.3 ± 0.1
6	YNYA	101.33	25.72	9.1 ± 0.3	0.8 ± 0.1	0.5 ± 0.1	2.8 ± 0.3	1.4 ± 0.1	0.8 ± 0.1
7	YNCX	101.49	25.05	8.6 ± 0.3	0.9 ± 0.1	0.3 ± 0.1	3.7 ± 0.3	1.5 ± 0.1	0.7 ± 0.1
8	XIAG	100.26	25.61	9.2 ± 0.4	0.7 ± 0.2	0.8 ± 0.2	2.9 ± 0.4	1.1 ± 0.2	0.8 ± 0.2
9	YNJD	100.88	24.44	10.3 ± 0.4	1.6 ± 0.1	0.9 ± 0.2	2.8 ± 0.4	1.4 ± 0.1	0.9 ± 0.2

**Table 2:** Variances of the detrended GPS time series corrected with rCMEs and oCMEs.

Station Name	East Component (mm^2^)			North Component (mm^2^)			Up Component (mm^2^)		
	$\sigma_{\mathrm{dGPS}}^{2}$	$\sigma_{rCME}^{2}$	$\sigma_{oCME}^{2}$	$\sigma_{\mathrm{dGPS}}^{2}$	$\sigma_{rCME}^{2}$	$\sigma_{oCME}^{2}$	$\sigma_{\mathrm{dGPS}}^{2}$	$\sigma_{rCME}^{2}$	$\sigma_{oCME}^{2}$
**YNYL**	6.4	3.7 ^#0^	3.8 ^#0^	3.4	1.6	2.1	75.1	41.3 ^#0^	44.2 ^#0^
**YNZD**	12.5	10.4	11.4	11.2	8.0 ^#0^	7.8 ^#0^	49.7	27.1	30.0
**YNLJ**	3.1	1.2	1.7	2.8	1.2	1.6	27.1	11.5	13.7
**YNYS**	4.4	2.3	2.8	2.7	0.9	1.3	33.5	15.7	18.0
**SCPZ**	3.7	1.7	2.0	2.4	1.2	1.6	30.6	13.5	15.8
**YNYA**	3.9	2.2	2.9	2.9	1.3	1.7	36.1	17.1	20.3
**YNCX**	4.3	2.0	2.3	2.6	1.2	1.6	44.3	16.0	18.7
**XIAG**	18.7	16.6 ^#1^	12.1 ^#1^	10.5	10.3 ^#1^	7.3 ^#1^	74.1	74.7 ^#1^	51.2 ^#1^
**YNJD**	15.6	14.2 ^#1^	12.7 ^#1^	7.2	5.1 ^#1^	4.5 ^#1^	64.4	45 ^#0^	42.3 ^#0^

$\sigma_{\mathrm{dGPS}}^{2}$ denotes variance of detrended GPS time series. The superscripts with #0 denote that variances are statistically equal to each other after rCME and oCME corrections, while those with #1 denote that variances with oCME correction are less than those with rCME correction. For the remainder, the variances with rCME correction are less than those with oCME correction.

**Table 3:** Root mean square (RMS) reductions of CME corrections for GPS time series.

Station Name	East RMS Reduction (%)		North RMS Reduction (%)		Up RMS Reduction (%)	
	rCME	oCME	rCME	oCME	rCME	oCME
**YNYL**	24.4	23.3	43.8	36.6	43.9	42.1
**YNZD**	9.5	6.0	20.5	21.3	42.5	39.4
**YNLJ**	35.5	23.2	43.3	36.2	56.8	53.0
**YNYS**	28.5	20.4	49.7	41.5	56.5	53.4
**SCPZ**	32.6	27.5	34.5	27.8	54.7	51.5
**YNYA**	25.0	13.4	41.9	34.0	54.5	50.4
**YNCX**	30.0	25.9	43.0	36.6	54.7	51.7
**XIAG**	5.8	19.6	3.4	18.4	20.4	34.1
**YNJD**	4.2	9.3	25.5	30.0	38.5	40.4

**Table 4:** Comparison the RMS reduction and Nash–Sutcliffe efficiencies (NSEs) between rCMEs and loading deformation for three components.

	RMS Reduction (%)				NSE			
	H	A	O	C	H	A	O	C
East	0.02	−0.08	−1.38	−1.38	−0.001	−0.003	−0.03	−0.03
North	10.53	0.04	0.60	12.82	0.20	−0.0001	0.01	0.24
Up	15.19	7.47	0.66	33.40	0.28	0.14	0.01	0.55

Note: H, A, and O denote HYDL, NTAL, and NTOL, respectively, and C denotes the combination of H, A, and O.
